# Evaluation of serum prolidase activity and oxidative stress markers in men with BPH and prostate cancer

**DOI:** 10.1186/s12894-017-0303-6

**Published:** 2017-12-12

**Authors:** Faruk Kucukdurmaz, Erkan Efe, Ahmet Çelik, Hasan Dagli, Metin Kılınc, Sefa Resim

**Affiliations:** 1 Department of Urology, Sutcu Imam University, Avsar Kampusu, 46100 Kahramanmaras, Turkey; 2 Department of Biochemistry, Sutcu Imam University, Kahramanmaras, Turkey

**Keywords:** Prostate cancer, Benign prostatic hyperplasia, Prolidase, Oxidative stress

## Abstract

**Background:**

Prostate cancer (PCa) and benign prostatic hyperplasia (BPH) are diseases of elderly men and are related to increased oxidative stress (OS). Although prolidase has a role in collagen metabolism, it is also used to evaluate OS in many diseases. However, there is a lack of data about serum prolidase activity (SPA) in prostate cancer. The aim of this study was to evaluate and compare SPA levels in males with BPH and PCa.

**Methods:**

Evaluation was made of a total of 81 men who underwent transrectal ultrasound guided prostate biopsy for a definitive diagnosis due to high PSA levels. Patients were separated into 2 groups as BPH and PCa patients. Pre-biopsy malondialdehyde (MDA), superoxide dismutase (SOD), PSA levels and serum prolidase activities (SPA) were compared between the groups and the correlations of SPA with the other parameters were also investigated in both groups.

**Results:**

BPH was diagnosed in 51 patients and PCa in 30. The mean age of patients was similar in both groups as 63.25 ± 5.81 years in the BPH group 65.30 ± 7.35 years in the PCa group(p:0.081). The median MDA and SOD levels were insignificantly increased in the PCa patients. SPA values were similar in BPH and PCa patients. SPA did not correlate with age, PSA, MDA or SOD levels in either group.

**Conclusions:**

Our study results revealed that serum prolidase activity is similar in BPH and PCa cases and is not correlated with MDA, SOD or PSA levels.

## Background

Prostate cancer (PCa) is the most common non- skin cancer and one of the main reasons for cancer- specific deaths in men [1]. Prostate cancer is a major public health problem in western countries, especially in the elderly population. Since life expectancy and the prevalence of PCa have increased, there is also expected to be an increased economic burden of the disease [2].

Benign prostatic hyperplasia (BPH) is a progressive disorder that develops secondary to increased proliferation of stromal and glandular cells, with a predominance of mesencyhmal cells [3]. BPH is characterised by the proliferation of stromal cells and increased extracellular matrix (ECM) deposition involving collagens, glycoproteins and proteoglycans. Cellular proliferation in the prostate is mainly modulated by age- associated changes in endocrine factors, although the exact pathophysiological mechanisms of BPH are not yet fully understood [4, 5].

PCa and BPH are the leading urological problems of aging men and manifest with disturbing obstructive and irritative symptoms. In addition to androgen and age dependence, oxidative stress, infection and inflammation are the other accepted predisposing factors for BPH and PCa [6, 7].

Oxidative stress (OS) is defined as the imbalance between the production and detoxification of reactive oxygen species (ROS). Those species can be involved in tumorigenesis by inducing genomic damage, tumor angiogenesis and augmentation of tumor cell migration [8]. It has also been reported that OS has a pivotal role in the aging process and disorders of aging males include BPH and PCa [9]. Malondialdehyde (MDA) is the principal end product of the lipid peroxidation pathway and is used as a marker to reflect oxidative status in normal subjects, BPH and PCa patients [5, 6, 9]. Superoxide dismutase (SOD) is the main endogenous antioxidant enzyme that counteracts the deleterious effects of ROS. Previous studies have reported that MDA levels are elevated and SOD activity is reduced in BPH and PCa patients compared to control groups [6, 9].

Matrix metalloproteinases (MMPs) are enzymes which are involved in in the degradation of ECM into proline and hydroxyproline [10]. Of these, prolidase is a manganese-dependent exopeptidase which cleaves to imidodipeptides and imidotripeptides with a C- terminal proline or hydroxyproline [11]. It is involved in the last stage of collagen turnover and, therefore, has a pivotal role in collagen metabolism, matrix remodelling and cell growth [10]. In addition, Yildiz et al. noted that serum prolidase activity (SPA) is directly correlated with oxidative stress and can be used as an OS marker [12]. Prolidase activity has been reported to be elevated in many tumor types including those in the kidney and bladder [13, 14]. Those studies also established that MDA levels were increased and SOD activities were reduced in men with bladder and kidney cancers. However, in both cancer types, prolidase activity was not found to be correlated with MDA concentrations and SOD activities.

Recently, prolidase activity has been investigated in men with BPH and has been reported to be significantly higher than that of control subjects [15]. However, there is a lack of data about SPA in PCa cases.

The objective of this reasearch was to compare MDA concentrations, SOD and prolidase activities of BPH and PCa patients. The associations between SPA, oxidative stress and antioxidant enzyme activities were also evaluated in both patient groups.

## Methods

This study was conducted in Kahramanmaras Sutcu Imam University, Turkey. Informed consent was obtained from each participant and the study protocol was approved by the Clinical ResearchEthics Committee of Kahramanmaras Sutcu Imam University (2016/20–257). All procedures performed in the study were in accordance with the ethical standards of the institutional and/or national research committee and with the 1964 Helsinki declaration and its later amendments.

### Subjects

The study population of this prospective research comprised patients who applied to our Urology Department between January 1 and June 1, 2017 with the complaint of lower urinary tract sypmtoms (LUTS). Participants were assessed using a comprehensive medical history and the International Prostate Symptom Score (IPSS) questionnaire to determine the symptom severity, measurement of peak urinary flow rate (Qmax; Aymed, Istanbul, Turkey) and digital rectal examination (DRE). Prostate specific antigen (PSA) testing was also performed for each patient. Patients who were over 40 years of age, with LUTS and a PSA value higher than the age-adjusted levels were enrolled in the study. This cohort comprised 130 males with PSA values higher than the age-adjusted levels and who underwent transrectal ultrasound (TRUS) guided prostate biopsy for a definitive diagnosis of either BPH or PCa. Exclusion criteria were determined as the presence of urinary tract infection, hematuria, previous medical or surgical intervention for prostate, history of previous prostate biopsy, diagnosis of any malignancy, renal and liver diseases, the use of antiinflammatory drugs, alcohol intake and antioxidant supplementation. When those criteria were applied, 49 patients were excluded and the study evaluation was made with a total of 81 subjects. At the beginning of the study, all cases were classified under two categories according to the pathology results as BPH or PCa. Patients diagnosed with PCa were also divided into subgroups according to the Gleason scores as Gleason = 6, Gleason = 7 and Gleason > 7. Prior to prostate biopsy, blood samples were obtained from each patient for SPA, MDA and SOD assays. These parameters were compared between the BPH and PCa subjects. Correlation of SPA with age, MDA, SOD and PSA was also investigated in both groups.

### Laboratory analysis

The day before the prostate biopsy, blood samples were obtained between 09:00–11:00 a.m. from each patient following 12-h fasting. The venous blood samples were collected into heparinized tubes and centrifuged at 3000 rpm for 10 min. The separated serum samples were stored at −80 °C until the day of laboratory investigation of MDA, SOD and SPA.

### Measurement of MDA

Plasma MDA levels were measured according to the Buege and Aust method [16]. MDA interacts with thiobarbiturate to give a red compound absorbing at 532 nm. The stock reagent (15% *w*/*v* trichloroacetic acid, 0.375% w/v thiobarbituric acid, 0.25 mol/l hydrochloric acid and 0.01% butylated hydroxytoluene) was thoroughly mixed with the sample and heated for 15 min in a boiling water bath. Then the sample was cooled and the precipitate was removed by centrifugation at 1000 x g for 10 min and the absorbance of the supernatant was determined at 532 nm against a blank containing all the reagents. The breakdown product of 1.1.3.3-tetraethoxypropane was used as the standard. MDA levels were calculated using 1.56 × 10–5 mol-1 cm-1 as the molar extinction coefficient. The results of the MDA measurements were given as nmol/ml.

### Measurement of SOD activity

SOD activity was measured using the Fridovich method [17]. The principle is based on the inhibition of nitro blue tetrazolium chloride (NBT) reduction by the xanthine-xanthine oxidase system, which is a superoxide generator. Xanthine (Sigma-Aldrich) and xanthine oxidase (Sigma-Aldrich) generate superoxide radicals that interact with NBT (SigmaAldrich) to produce a red formazan dye. SOD activity is then measured according to the level of inhibition of this reaction. One unit of SOD was defined as the quantity of enzyme (mg) causing 50% inhibition in the NBT reduction rate. SOD activity was stated as U/ml.

### Measurement of serum prolidase activity

Serum was diluted 40-fold with 2.5 mmol/L Mn2+ and 40 mmol/L Trizma HCl buffer (pH 8.0) and preincubated at +37 °C for 2 h. The resultant mixture which contained 30 mmol/L gly-pro, 40 mmol/L Trizma HCl buffer (pH 8.0), and 100 μL of preincubation serum in 1 mL was incubated at +37 °C for 30 min. The incubation reaction was stopped after the addition of 0.5 mL of 20% trichloroacetate solution. The supernatant was used for determination of proline with the Myara method which was modified from the Chinard technique [18]. All reagents were of analytical grade and purchased from Sigma (St. Louis, Missouri, USA) and Merck (Darmstadt, Germany). Intra and interassay precision performances of the assay were determined from a serum pool on 10 replicates in a single run and in 10 different runs, respectively, and yielded CV of 3.8 and 9.0%.The resultswere reported as U/L.

### Statistical analysis

Analyses of the study data were performed using SPSS® 21.0 for Windows software.

The data were presented as mean ± standard deviation (SD), median, minimum and maximum values. Parametric continuous variables were compared using the Student’s t test. Pearson’s correlation test was used for correlation analyses. A value of *p* < 0.05 was accepted as statistically significant.

## Results

Of the total 81 cases, BPH was diagnosed in 51 and PCa in 30 according to the pathology results of prostate biopsies. Of the 30 PCa patients, 12 were determined as Gleason = 6, 12 as Gleason = 7 and 6 as Gleason >7. The demographic and laboratory features of the groups are given in Table 1.

**Table 1:** Demographic and laboratory parameters of the study population

	BPH (*n* = 51)	PCa (*n* = 30)	*p* value
Age	63.25 ± 5.81	65.30 ± 7.35	0.081
BMI (kg/m^2^)	22.13 ± 1.28	22.74 ± 2.11	0.252
IPSS	17.21 ± 4.82	18.53 ± 5.12	0.121
PSA	6.20 (3.28–40.30)	13.24 (5.19–390.81)	0.025^a^
MDA	36.77(21.29-56.89)	38.7 (25.16–146.29)	0.075
SOD	19.44 (1.5–278.4)	40.42 (3.13–110.79)	0.512
SPA	1939 (1242–2722)	1845 (445–3244)	0.342

*BMI* Body mass index Hypertension, diabetes mellitus,*IPSS* International prostate symptom score, *PSA* Prostate specific antigen, *SOD* Superoxide dismutase, *MDA* Malondialdehyde, *SPA* Serum prolidase activity

Data were given as median (min-max) or mean ± standart deviation

^a^statistical significance

The mean age of the patients was similar in both groups as 63.25 ± 5.81 years in BPH group and 65.30 ± 7.35 years in PCa group (p:0.081). The mean body mass index (BMI) values were similar in both groups (22.57 ± 1.25 vs. 23.11 ± 1.84, p:0.752). No statistically significant difference was determined between BPH and PCa patients in respect of the mean IPSS values (17.54 ± 5.41 vs. 18.71 ± 6.04, p:0.124) and the Qmax values (9.84 ± 2.15 ml/s vs 10.21 ± 2.84 ml/s, p:0.232). PSA values were statistically significantly higher in the PCa cases (*p* = 0.025). The median MDA and SOD levels were increased in the PCa group but not to a statistically significant level. Serum prolidase activities were found to be similar in the BPH and PCa patients.

**Fig. 1:**
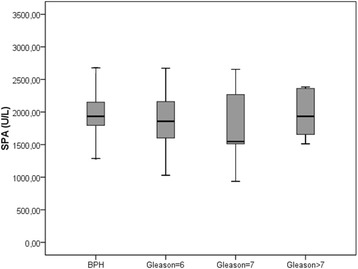
Serum prolidase activities in men with BPH and prostate cancer

PSA, MDA, SOD levels and prolidase activities in BPH and subgroups of PCa are given in Table 2. PSA levels were not significantly different between the BPH, Gleason = 6 and Gleason = 7 PCa patients. PSA values of the PCa patients with Gleason score > 7 were significantly higher than those of the other groups. There were no statistically significant differences between the groups in terms of MDA and SOD levels. SPA was found to be similar among the study groups (Fig. 1).

**Table 2:** Comparison of the age, oxidative stress, antioxidant enzyme and serum prolidase activity parameters according to pathology results of the patients

	BPH	Gleason = 6	Gleason = 7	Gleason > 7	*P*
SPA (IU)	1939 (1242–2722)	1872 (445–3244)	1548(864–2619)	1905(1512–2385)	0.330
PSA (ng/ml)	6.2(3.28–40.3)	9.3(6.6–38.0)	13.4(5.1–116.8)	55.3(6.4–390.8)	0.001^a^
MDA (nmol/ml)	36.76(21.2–56.8)	42.1(26.7–146.2)	38.7(25.1–53.0)	46.4(36.7–59.9)	0.078
SOD (u/ml)	19.43 (1.50–278.4)	37.3(3.1–84.4)	49.0(8.2–104.3)	39.2 (7.3–110.7)	0.647

Data were given as median (min-max), *SPA* Serum prolidase activity, *PSA* Prostate specific antigen, *MDA* Malondialdehyde, *SOD* Superoxide dismutase

^a^statistical significance

The correlations of serum prolidase activities with age, PSA, MDA and SOD levels in males with BPH and PCa are presented in Table 3. In both groups, SPA did not show any correlation with age, PSA, MDA or SOD levels.

**Table 3:** Correlations of age, PSA, oxidant and antioxidant parameters with serum prolidase activity in men with benign prostatic hyperplasia and prostate cancer

	BPH	PCa		
	r	*p* value	r	*p* value
Age	−0.097	0.50	0.060	0.766
PSA	0.175	0.22	−0.045	0.825
MDA	−0.026	0.859	0.026	0.897
SOD	−0.108	0.45	−0.250	0.208

*r* Pearson correlation coefficient, *PSA* Prostate specific antigen, *MDA* Malondialdehyde, *SOD* Superoxide dismutase

## Discussion

In the present study, SPA, MDA and SOD levels in men with BPH and PCa have been evaluated. Also the correlation of SPA with age, PSA and other parameters was investigated. To the best of our knowledge, this is the first study to have compared SPA in BPH and PCa cases diagnosed from prostate biopsy due to high PSA levels.

BPH and PCa are diseases of elderly males which both present with LUTS and the differential diagnosis for BPH and PCa is made by using PSA and DRE. Any suspicious lesion on DRE or a PSA value higher than the age-adjusted levels may necessitate the use of a TRUS-guided prostate biopsy for histopathological diagnosis.

The interactions between ECM components, cellular compartments and endocrine features render the role of ECM pivotal in the pathogenesis of epithelial abnormalities, including BPH and cancer [19]. Collagen is the main component of ECM in many tissues including prostate. Histochemical and morphometric analyses have established 3 major collagen types in human prostate as types I, III and IV. BPH is characterised by a change in collagen content (elevation of types I and IV, and a reduction in type III collagen) and an increase in ECM deposition [4]. The progression of cancer depends on the degradation of collagen and other ECM components which are mediated through proteolytic enzymes released from the cancer cells. MMPs are the enzymes necessary for the cleavage of ECM proteins which serve as a barrier to the invasion of tumor cells [20]. Prolidase, one of the MMP enzymes, is a homodimeric iminopeptidase which releases a carboxy- terminal proline or hydroxyproline from oligopeptides and is involved in collagen turnover and cell growth [11]. It regulates the last stage of the collagen breakdown. It has also been suggested to be the rate limiting factor of collagen metabolism. Since it has been suggested that the invasion and spread of cancer cells depend on the degradation of ECM, it can be hypothesized that prolidase activity can be associated with the invasiveness and aggressivity of the cancer [20]. Although serum prolidase activity has been well established in other cancer types including the kidney, bladder and ovaries [13, 14, 21], there are no data in literature about its activity in prostate cancer.

The results of the current study revealed that serum prolidase activity was reduced in PCa patients when compared to the BPH group, although the difference was not statistically significant. This may be due to the increased collagen turnover in patients with BPH as defined by Gecit et al. [15]. In addition, it was established that prolidase levels were not correlated with the PSA levels of PCa patients.

It has been reported that prolidase activity is elevated in bladder and kidney cancers. However, the findings obtained in the current study were similar to those of Palka et al., where prolidase levels were found to be lower in pancreatic cancer patients [22].

Oxidative stress, by way of ROS, may be associated with the production of DNA adducts indirectly by inducing autocatalytic lipid peroxidation which results in the formation of several degradation products such as malondialdehyde (MDA) and alkoxyl radicals. MDA, the final product of lipid peroxidation of polyunsaturated fatty acids, is used as a non-invasive biomarker of OS to evaluate radical-mediated pathophysiological conditions [23]. SOD, CAT and glutathione peroxidase (GPX) are the antioxidant enzymes involved in the endogenous defense mechanisms against increased ROS. Of these, SOD can be used to reflect the antioxidant activity in patients with BPH and/or PCa [6, 9]. Recently, prolidase has been shown to have a strong relationship with OS and is thought to be a novel marker to demonstrate the oxidative status of subjects in many disorders [24, 25].

Aging and subsequent OS are the common, recognized risk factors for BPH and PCa [7]. OS can produce vascular tissue damage, genomic damage and post-translational DNA alterations that are involved in DNA repair and apoptosis. Since the human prostate is subject to OS damage due to rapid cell turnover and fewer DNA repairing mechanisms, those events may result in point mutations, deletions or rearrangements which may lead to either benign hyperplasia or prostate cancer [26]. The relationship between OS, antioxidant enzymes and BPH has been evaluated in several reports with conflicting outcomes. Savas et al. reported no significant OS differences between men with BPH and a control group [27]. In contrast, Aydın et al. demonstrated increased MDA and a reduced antioxidant enzyme level in men with BPH [6]. More recently, Gecit et al. stated that men with BPH had higher OS when compared to control subjects [15]. It was also noted that prolidase activity was associated with increased OS parameters in BPH subjects.

Previous studies have shown that increased OS and reduced antioxidant enzyme levels were significant in PCa cases compared to BPH and control subjects [6, 9]. Since MDA is used to estimate OS in tissues, the use of MDA in the diagnosis of PCa has also become more prevalent. The MDA levels of PCa men have been reported to be significantly elevated when compared to BPH cases [9]. However, Dogru-Abbasoglu reported that serum lipid peroxide levels were not discriminative between BPH and PCa patients [28]. In the present study, it was determined that MDA concentrations were insignificantly increased in all PCa groups when compared to the BPH group and the differences in the MDA levels of the PCa patients were not correlated with the Gleason score.

Studies evaluating SOD enzyme activities in men with benign and malignant prostatic tissues have revealed that PCa patients had higher MDA and SOD levels compared to control subjects [29, 30]. In the present study, PCa patients had insignificantly higher SOD activities than BPH cases. Since the MDA levels were also insignificantly increased in men with PCa, it can be speculated that antioxidant enzymes might be increased in response to increased OS to nullify its deleterious effects.

Although it has been reported that prolidase activity is strongly related to OS, the results of the current study showed no associations between serum prolidase activity, MDA levels and SOD activities in BPH and PCa patients. In addition, prolidase activities were not correlated with the PSA values of the patients. This may have been due to the relatively small number of the patient population or the lack of knowledge about the factors related to the activity of prolidase in prostatic diseases. Therefore, further large scale studies are required to support these preliminary findings. In addition to the relatively small patient population, other limitations of the current study can be considered to be that MDA, SOD and serum prolidase activities in the prostatic tissues were not studied, other markers such as total antioxidant status (TAS), total oxidant status (TOS), NO level, vitamins (C and E), glutathione peroxidase (GPx), glutathione-S-transferase (GST) were not examined and metastatic evaluation of PCa patients was not performed.

Although prolidase has a pivotal role in collagen biosynthesis and has been suggested as a marker of OS, data about its activity in different cancer types and disorders known to be associated with increased OS have been conflicting. Therefore, prolidase should be combined with other biochemical markers in order to provide more accurate data to clinicians about the investigated disease activity.

## Conclusions

BPH and PCa are diseases of aging men and are associated with increased OS. Although prolidase was thought to be a marker of OS in BPH, the results of this study failed to present any association between prolidase activity and OS in both patient groups. Prolidase activity was also not found to be related to PSA levels in either the BPH or PCa patients.

## Abbreviations

BPH: Benign prostatic hyperplasia
DRE: Digital rectal examination
ECM: Extracellular matrix
LUTS: Lower urinary tract symptoms
MDA: Malondialdehyde
MMPs: Matrix metalloproteinase
OS: Oxidative stress
PCa: Prostate cancer
PSA: Prostate specific antigen
SOD: Superoxide dismutase
SPA: Serum prolidase activity
